# Antibacterial Activity of New Shigaite-like Ni/Co–Al and Ni/Cu–Al Layered Double Hydroxides Intercalated with Sulfate and Sodium Cations: Preliminary Studies

**DOI:** 10.1021/acsomega.6c02816

**Published:** 2026-06-12

**Authors:** Anne Raquel Sotiles, Vitor Vianna de Souza Machado, Monica Surek, Cláudia Eliana Bruno Marino, Fernando Wypych

**Affiliations:** † Department of Chemistry, Centro Politécnico, Federal University of Paraná, CP 19032, Jardim das Américas, 81531-980 Curitiba, Paraná, Brazil; ‡ Department of Mechanical Engineering, Program in Engineering and Materials Science (PIPE), Centro Politécnico, Federal University of Paraná, CP 19011, Jardim das Américas, 81531-980 Curitiba, Paraná, Brazil; § Department of Clinical Analysis, Campus Jardim Botânico, Federal University of Paraná, Jardim Botânico, 80210-170 Curitiba, Paraná, Brazil; ∥ Federal University of Technology − Paraná, Avenida dos Pioneiros, 3131, 86036-370 Londrina, Paraná, Brazil

## Abstract

Solid solutions of bimetallic and trimetallic layered double hydroxides (LDHs) containing aluminum and cobalt or aluminum and copper, with the chemical compositions [Ni_6–_ *_x_* M_*x*_^2+^Al_3_(OH)_18_]­[(SO_4_)_2_Na]·*y*H_2_O (M^2+^ = Co or Cu and *x* varied from 0 to 6), were synthesized by coprecipitation with increasing pH at room temperature, hydrothermally treated at 90 °C for 120 h and dried at 60 °C for 48 h. The compounds were analyzed by X-ray diffraction (XRD), whereby all systems presented basal distances close to 11 Å, typical of sulfate intercalation in double-layer arrangements in the presence of hydrated alkali metal cations. FTIR spectra indicated the presence of typical bands attributed to sulfate, water molecules and metal–oxygen vibrations (M–O). Scanning electron microscopy (SEM)/energy-dispersive spectroscopy (EDS) and ICP/OES also corroborated the successful synthesis of all LDH solid-state solutions. The preliminary potential antibacterial activity of the LDHs was evaluated using the agar well diffusion method against *Staphylococcus aureus* (*S. aureus*) and *Escherichia coli* (*E. coli*), while the bimetallic CuAl-LDHs had the most effective antibacterial activity.

## Graphical abstract

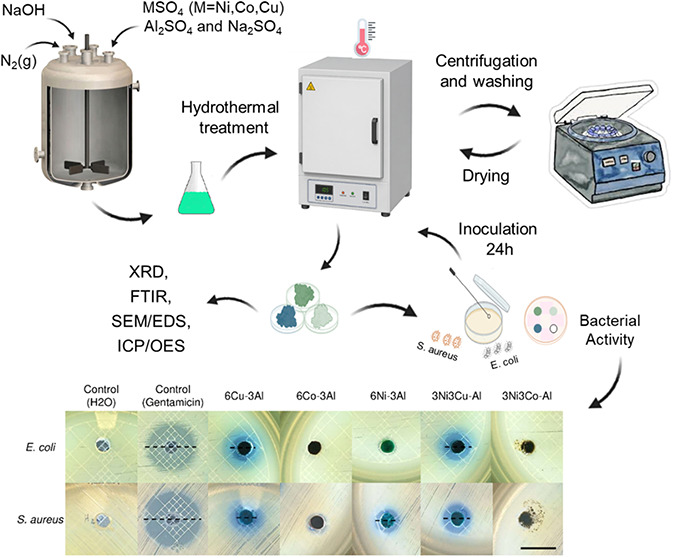

## Introduction

Traditional layered double hydroxides (LDHs) are represented with the generic chemical formulation [M_1–*x*_^2+^M_*x*_^3+^(OH)_2_]­(A*^n^* ^–^)_*x*/*n*_·*y*H_2_O, where M^2+^ denotes a divalent cation occupying octahedral centers coordinated with hydroxyl anions and M^3+^ represents the trivalent cations that partially isomorphically replace the M^2+^ cations in the brucite-like (Mg­(OH)_2_) structure, generating an excess of positive charges in the layers.

These positively charged layers, obtained by edge-sharing octahedra, are neutralized with negatively charged anions [(A^*n*–^)_*x*/*n*_·*y*H_2_O]^*x*−^, which are intercalated between the layers. The divalent and trivalent cations must have close ion radii to avoid distortions in the M–O bonds, which can hinder obtaining long-range crystalline domains.

Several excellent reviews related to diverse applications of LDHs have been published so far.^1–7^ An emerging field involves LDHs’ use as active material for antibacterial,^8–16^ antifungal,^17,18^ and antifouling (antibiofilm) applications.^19–25^ Most of the time, the active anions have been intercalated between the layers, so the activity rarely comes specifically from the inorganic layers, as proposed in the present study.

Copper has been traditionally used as a flexible material for antimicrobial purposes, such as on surfaces, on nanoparticles, and in solutions, to prevent the spread of pathogens in agriculture and healthcare. Copper ions act through multiple mechanisms, variously causing lipid peroxidation, protein oxidation, and DNA damage. The metal ions inside the cells damage the DNA and affect the ATP production.

Furthermore, they can interact with phosphate and −SH groups of proteins and DNA, causing denaturation and other structural perturbations.^26^ In the case of cobalt, in both the bi (6Co-3Al) and trimetallic (3Ni/3Co-3Al) phases, inhibition has been limited only to *Staphylococcus aureus* (*S. aureus*) studied the synthesis of M^2+^Al LDHs (M^2+^ = Mn, Mg, Ni, Cu, and Co), and the inhibition capacity of Cu/Al LDH is relatively higher than the activity of Ni/Al and Co/Al against *Escherichia coli* (*E. coli*), but that Co/Al LDH may excel against *S. aureus*.^27^ The antibacterial activity of LDHs has been attributed to the M^2+^ present in the structure, while the aluminum cations are inactive,^28,29^ and functionalized bone cement with a traditional Mg/Cu–Fe trimetallic LDH and a survival rate of *S. aureus* below 0.01% was observed, indicating high antibacterial activity. Another Mg/Al LDH was synthesized and incorporated with Cu_2_O particles and tested against strains of *Bacillus cereus* and *Pseudomonas stutzeri*. Both studies attributed the inhibition effect to the action of copper, which induces oxidative stress and disrupts bacterial membranes, causing structural damage.^29,30^

A trimetallic LDH containing Zn/Cu–Al was also synthesized and its potential bactericidal activity was tested against *E. coli* and *S. aureus*, showing positive effects, but lower than when the sample was calcined at 400, 600, and 800 °C. The authors attributed the action to the presence of copper and its availability, since at these temperatures the material is unstructured and the metal is more available to diffuse into the culture medium gel.^31^

Various mechanisms have been reported in the literature, such as the release of metal ions into the medium, which can diffuse into bacterial cells, affecting amino acid metabolism and the enzyme system, or binding to intracellular bacterial DNA and proteins, leading to their denaturation or deactivation. Furthermore, LDHs can act through electrostatic interaction in the case of Gram-negative bacteria since the surface of the material is positively charged. Hydroxide ions are strong oxidizing species that can cause protein denaturation and damage to the bacterial DNA or the cytoplasmic membrane.

They also absorb water to create hydroxyl radicals, and in the presence of oxygen, they generate superoxide ions that damage bacterial cells.^27,32^

However, in general for both Gram-negative and Gram-positive bacteria, the presence of LDH can reduce nutrients in the medium through adsorption and affect bacterial growth, in addition to adhering to the bacterial wall, leading to the blockage of nutrient transport channels and inhibiting growth.^24,25,27,33^

Recently, we described a new class of layered double hydroxides with the chemical formulations [M_6_^2+^M_3_^3+^(OH)_18_]­[B^+^(H_2_O)_6_(X^2–^)_2_]·6H_2_O (M^2+^ = Mg, Mn, Zn, Co, Ni,Cu; M^3+^ = Al, Cr; B^+^ = Li, Na, K, NH_4_; X^2–^ = SO_4_, HPO_4_).^34–37^ These materials are analogous to the minerals, viz., motukoreaite (M^2+^ = Mg), natroglaucocerinite (M^2+^ = Zn), shigaite (M^2+^ = Mn), and nikischerite (M^2+^ = Fe), as well as to sulfate green rusts, denoted GR_SO4_, which are mixed-valent iron-bearing LDHs containing sulfate and alkali metal cations (NaFe_6_^2+^Fe_3_^3+^(SO_4_)_2_(OH)_18_·12H_2_O, KFe_6_^2+^Fe_3_^3+^(SO_4_)_2_(OH)_18_·12H_2_O).^38–45^

The novelty of these compounds is the possibility of exchanging not only anions but also cations and even both simultaneously. Also, this class of compounds has been described as trimetallic phases containing two divalent metals in the presence of aluminum.^46,47^ As far as we know, the only article involving the antibacterial activity using a Pickering emulsion stabilized with sodium natroglaucocerinite intercalated with Ag^+^ and decorated with Ag^0^/Ag_2_O nanoparticles was that of Amaral et al.^48^

To contribute to this field of research, the objective of the present work is to describe the synthesis and characterization of several new bimetallic and trimetallic compounds with the chemical composition [Ni_6–_ *_x_* M_*x*_^2+^Al_3_(OH)_18_]­[(SO_4_)_2_Na]·*y*H_2_O (M^2+^ = Co or Cu and *x* varied from 0 to 6) and investigate their potential antibacterial activity, especially by releasing metal cations from the layers despite the very low solubility of the layered compounds.^49,50^

## Materials and Methods

Bimetallic layered double hydroxides (LDHs) were synthesized with an M^2+^/Al^3+^ ratio of 2:1, where M^2+^ = Ni, Co, or Cu, while trimetallic LDHs were synthesized with the formula [Ni_6–_ *_x_* M_*x*_^2+^Al_3_(OH)_18_]­[M^+^(H_2_O)_6_(SO_4_)_2_]·6H_2_O (*x* from 0 to 6, M^2+^ = Co and Cu and M^+^ = Na), in both cases by coprecipitation at increasing pH levels.

A solution containing the mixture of metal sulfates (Table 1) was prepared with ultrapure and decarbonated water from a Milli-Q system (18.2 MΩ·cm, Millipore Simplicity UV) and was added to a glass reactor under stirring and pH control, with automatic titration (Up Control).

**Table 1:** Amounts of Chemicals Used (in mmol), Initial and Final pH during LDH Syntheses

compound	NiSO_4_	CoSO_4_	CuSO_4_	Al_2_(SO_4_)_3_	Na_2_SO_4_	initial pH	final pH
6Ni-3Al	25.639			6.409	2.142	3.43	7.54
5Ni/1Co-3Al	21.360	4.270		6.407	2.140	3.97	8.03
4Ni/2Co-3Al	17.085	8.544		6.406	2.350	3.91	8.17
3Ni/3Co-3Al	12.812	12.810		6.410	2.135	3.86	8.05
2Ni/4Co-3Al	8.540	17.06		6.404	2.135	3.98	8.01
1Ni/5Co-3Al	4.268	21.342		6.402	2.134	3.76	8.05
6Co-3Al		25.620		6.406	2.142	3.40	9.51
5Ni/1Cu-3Al	21.275		4.255	6.383	2.127	3.28	7.54
4Ni/2Cu-3Al	16.952		8.477	6.356	2.118	3.57	7.56
3Ni/3Cu-3Al	12.660		12.660	6.330	2.110	3.86	7.61
2Ni/4Cu-3Al	8.406		16.809	6.304	2.102	3.85	7.59
1Ni/5Cu-3Al	4.189		20.929	6.278	2.092	3.82	7.63
6Cu-3Al			25.108	6.271	2.089	3.13	8.19

The ideal conditions were determined in previous experiments where titration curves were obtained to determine the ideal precipitation pH, and the synthesis conditions were established in such a way that the growth particles prevailed instead of the nucleation process. Low saturation conditions are obtained using low concentrations of the reactants, constant vigorous stirring, and extremely low addition of the alkaline solution, which occurs over the internal N_2_ gas flow to avoid the presence of carbonate anions, a frequent contaminant in these syntheses.

The pH values of the bimetallic LDH phases Ni/Al, Co/Al, Cu/Al have been reported in previous studies,^35,51^ while for the trimetallic combinations, intermediate pH values between the simple phases were chosen, at pH values only slightly exceeding that of the Ni/Al phase. After the syntheses, the materials were removed from the reactor and hydrothermally treated in an oven at 90 °C for 120 h. The resulting solids were subsequently separated by centrifugation at 4500 rpm, washed several times with decarbonated distilled water and dried at 60 °C for 48 h. The combinations, proportions and pH values used in the synthesis of the compounds are presented in Table 1.

The X-ray diffraction (XRD) patterns were obtained using a Shimadzu XRD-6000 diffractometer with Cu Kα = 1.5418 Å radiation. After aging and the last centrifugation step, drops of the slurry were deposited on glass sample holders and dried at room temperature. The analyses were performed using tension of 40 kV and current of 30 mA, with a dwell time of 2° min^–1^.

The samples were also characterized by FTIR by using a Vertex 70 spectrophotometer. KBr pellets containing around 1% (w/w) of the LDH were gently mixed and pressed at 5 tons with a Shimadzu hydraulic press, and the spectra were collected in transmission mode by accumulating 32 scans in the region of 400–4000 cm^–1^, using a resolution of 4 cm^–1^.

The composition of the materials was evaluated by energy-dispersive spectroscopy (EDS) using a Tescan Vega3LMU microscope with AZ Tech software. The samples containing Ni and Co, after dispersion in water, were deposited on copper tapes and dried at room temperature. After EDS spectra were collected using 15 eV and an accumulation time of 45 s, the samples were sputter-coated with a thin layer of gold to obtain scanning electron microscopy (SEM) images using a Tescan Mira3 microscope. For samples containing copper, a carbon tape was used in the analysis.

The quantitative analyses of the metals and sulfur (relative to SO_4_^2–^) used to formulate the samples’ chemical composition were performed with a Thermo Scientific model iCAP 6500 ICP/OES spectrometer after dissolving the samples in 1.0% v/v of HNO_3_ in Milli-Q water solution. The data were collected in triplicate, treated with the Thermo Scientific iTeVa software version 1.2.0.30, and average values were used to obtain the LDHs’ compositions. The release of divalent cations in sodium chloride solution (3.5 wt %) were also measured for 6Ni-3Al and 6Cu-3Al LDHs after 1 h, 24 h and 7 days of powder immersion.

The antibacterial activities of the LDHs were evaluated by the agar well diffusion method.^52,53^ Briefly, the surface of the Mueller-Hinton agar plates (Bio-Rad, Marnes-la-Coquette, France) was inoculated using a sterile swab immersed in a saline dispersion containing 1 × 10^8^ CFU/mL of each bacterial strain: the Gram-positive bacterium *S. aureus* ATCC 6538 (*S. aureus*) or the Gram-negative bacterium *E. coli* ATCC 25922 (*E. coli*). Subsequently, wells were made in the agar (8 mm diameter), which were filled with 33 mg of each LDH and 100 μL of sterile distilled water. As positive control, 100 μL of a gentamicin solution (100 μg/mL) (Amresco, Solon, OH, USA) was used. The plates were incubated at 35 °C in ambient air for 24 h.^53^

In these assays, 100 μL of a previously prepared dispersion at a concentration of 100 mg/300 μL was used (33.33 mg of LDH). A fixed amount of LDH was employed to ensure the quantitative reliability of the results, considering the inherent uncertainties in determining the number of moles and the distinct solubility characteristics of each compound. The results are expressed as mean ± standard deviation of the complete bacterial inhibition zone diameters based on two independent experiments.

## Results and Discussion

XRD analysis of the Ni/Co and Ni/Cu sample groups (Figures 1 and 2) showed basal peaks characteristic of layered materials. The XRD patterns evidenced the presence of only basal peaks (00l) of LDHs due to the particle orientation resulting from the deposition method of the material on the sample holder.

**Figure 1:**
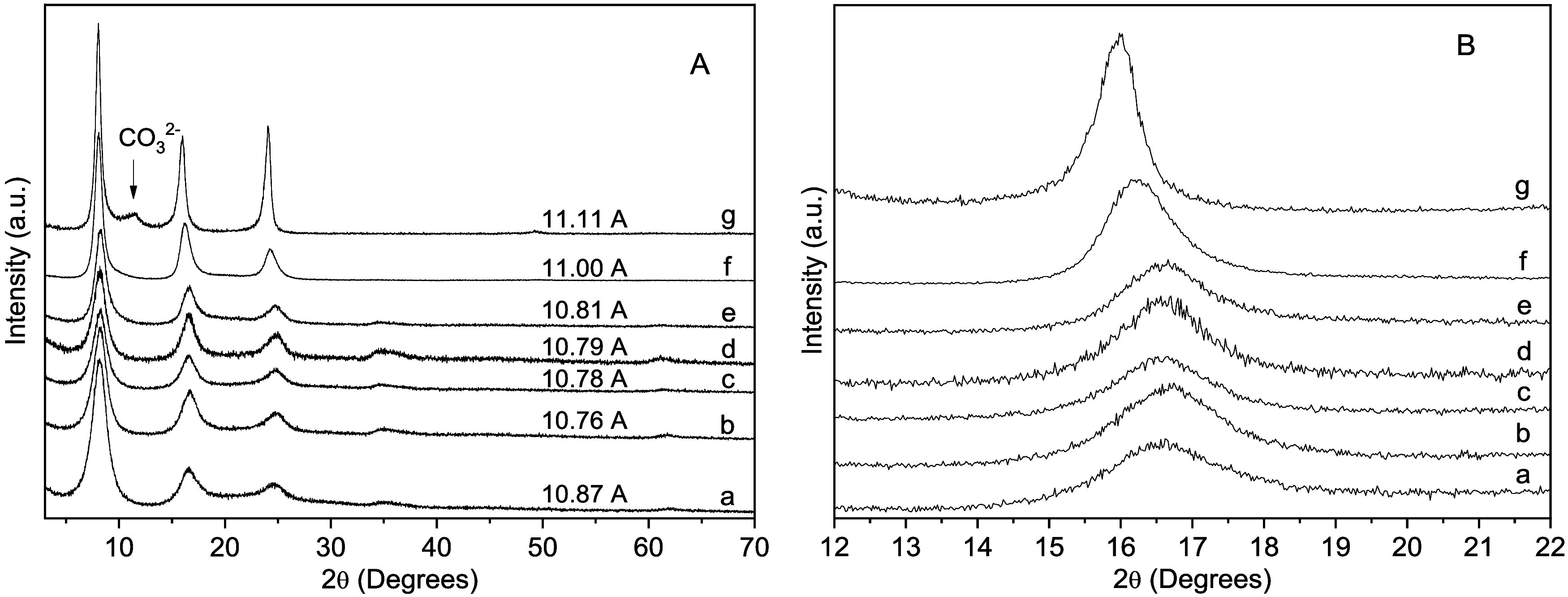
(A) X-ray diffraction patterns of the samples 6Ni-3Al (a), 5Ni/1Co-3Al (b), 4Ni/2Co-3Al (c), 3Ni/3Co-3Al (d), 2Ni/4Co-3Al (e), 1Ni/5Co-3Al (f), and 6Co-3Al (g). (B) Expansion of the XRD patterns in the region of the second basal peak.

**Figure 2:**
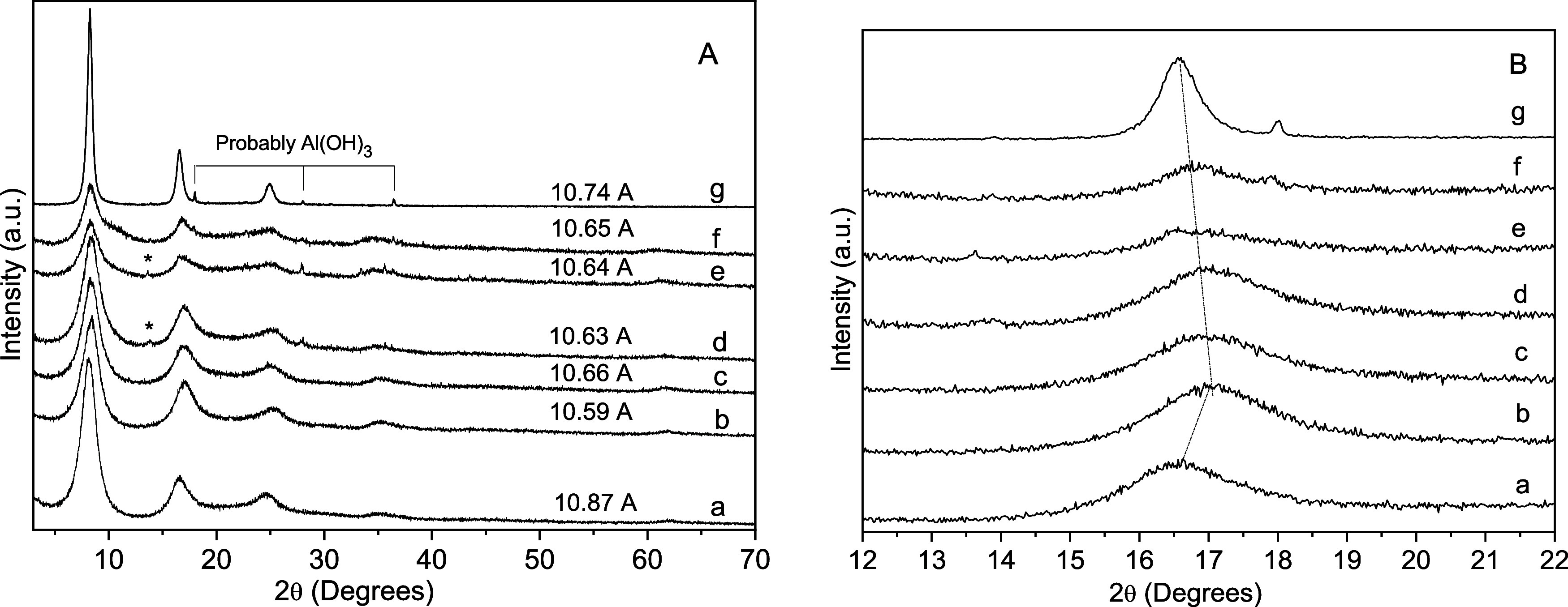
(A) X-ray diffraction patterns of the samples 6Ni-3Al (a), 5Ni/1Cu-3Al (b), 4Ni/2Cu-3Al (c), 3Ni/3Cu-3Al (d), 2Ni/4Cu-3Al (e), 1Ni/5Cu-3Al (f), and 6Cu-3Al (g). (B) Expansion of the XRD patterns in the region of the second basal peak. * Impurities.

The basal distance of the compounds was calculated from the third basal peak and presented values close to 11 Å, which is characteristic of intercalated sulfate in double-layer arrangement in the presence of sodium cations, both in hydrated form.

In the case of the Ni/Co sample group, the 6Ni-3Al sample presented a basal distance of 10.87 Å, and when the cobalt substitution process began, there was a reduction in the basal distance to 10.76 Å in the 5Ni/1Co-3Al sample (Figure 1-a,b). Subsequently there was a gradual increase in the basal distances with increase in the cobalt proportion until reaching 11.11 Å in the synthesis of the sample containing only cobalt (Figure 1-g). The sample 6Co-3Al also presented a small intensity peak attributed to one phase contaminated by carbonate ions, possibly due to the higher pH value used in the synthesis where carbonate prevails in the equilibrium CO_2_/HCO_3_^–^/CO_3_^2–^. In this same sample, the intensity of the third basal peak of the intercalated SO_4_^2–^/Na^+^ is intensified due to the superposition with the second basal peak of the intercalated CO_3_^2–^.

The increase in the basal distance in the series occurs due to the increase in the radius of the Co^2+^ cation (0.742 Å) that is replacing Ni^2+^, which has a smaller ionic radius (0.704 Å). This was also corroborated by the length of the M–O bonds for octahedral geometry, namely, 2.108 and 2.070 Å for cobalt and nickel, respectively.^54^ The increase in the basal distance can also be due to several features such as slight variations in the layer charge resulting from different M^2+^/Al^3+^ atomic ratios in the samples, different nature of the compensating anions such as the introduction of larger amount of CO_3_^2–^, the hydration degree of the sample, among others.

Samples containing only Co or with higher Co contents in relation to Ni presented higher crystallinity, as already reported in the synthesis of shigaite-like bimetallic LDH obtained with Co/Al and Ni/Al, all intercalated with sulfate and different alkaline cations.^51^

In the Ni/Cu/Al sample group (Figure 2), there was a decrease in basal spacing with the beginning of nickel substitution by copper, followed by an increase in the sample containing only copper (6Cu-3Al) (Figure 2-g).

Furthermore, even though copper has cations with larger ion radius (0.764 Å) than nickel (0.704 Å), the final phase synthesized with only copper (6Cu-3Al) had a smaller basal distance (10.72 Å) than the initial phase synthesized with only nickel (10.87 Å). This discrepancy in the behavior of the copper-containing samples is related to the fact that octahedral copper presents the Jahn–Teller (JT) distortion effect, with elongation of the axial bonds in relation to the equatorial bonds. Similar behavior was described in other trimetallic shigaite-type LDHs synthesized with Zn/Cu/Al and Co/Cu/Al^47^ and oxysalts.^55^

As the samples became richer in Cu, the presence of other diffraction peaks was detected, the first located close to the second basal peak of the LDH, in the 2θ region of 18°, with a basal distance close to 4.8 Å, likely attributed to Al­(OH)_3_. The other impurities could not be identified and Al­(OH)_3_ seemed to be present in very small concentrations. According to a study involving the synthesis of a Zn/Al LDH, the synthesis mechanism was described as initially involving the formation of Al­(OH)_3_ and the subsequent incorporation of the M^2+^ metal into the structure to obtain the LDH structures.^56^ We have no plausible explanation why the sample 6Cu-3Al (Figure 2-g) is more crystalline than the other samples, despite the Jahn–Teller effect is more pronounced in this sample.

The presence of Al­(OH)_3_ in the material could be explained by the incomplete incorporation of Cu into the structure, which could be a consequence of Jahn–Teller (JT) distortion, resulting in the difficult incorporation of the distorted octahedron together with the already formed Al­(OH)_3_. Some LDHs have already been obtained with copper in the bimetallic Cu/Al and trimetallic phases with Cu/Co/Al and Cu/Zn/Al with different intercalated anions,^47,57,58^ both in traditional and shigaite-like LDH structures, contradicting the idea that LDH phases containing Cu would be formed only in the presence of another M^2+^.^59^

In addition to the difference in sample crystallinity due to partial or full Ni substitutions, crystalline domain sizes along the basal direction (particles) were calculated using the Scherrer equation (*D*_p_ = (0.94 × λ)/(β × cos θ), where *D*_p_ = crystalline domain size, β = diffraction peak broadening in radians, θ = Bragg angle, and λ = X-ray wavelength), resulting in larger crystalline domain sizes for the 6Co-3Al sample than for 6Cu-3Al and 6Ni-3Al samples, with dimensions approaching 15, 10, and 5 nm, respectively. Furthermore, the number of packed layers was also higher in the sample containing only cobalt (∼13) in comparison to the sample containing only nickel (∼4). However, a trend toward increasing crystalline domain size and packed layers occurs as Ni was replaced by Cu (Supporting Information SM1).

The FTIR spectra of all phases of the Ni/Co and Ni/Cu group of samples (Figure 3) were consistent with the spectra already reported for bimetallic and trimetallic phases containing intercalated sulfate.^34,46,47^ These samples present a broad band in the region of 3400 cm^–1^, which is attributed to the stretching vibrations of the O–H bond of the LDH structure, in addition to physiosorbed/intercalated water molecules, along with a signal at 1630 cm^–1^ that is characteristic of the bending vibrations of the O–H bond of the water molecules.^60^

**Figure 3:**
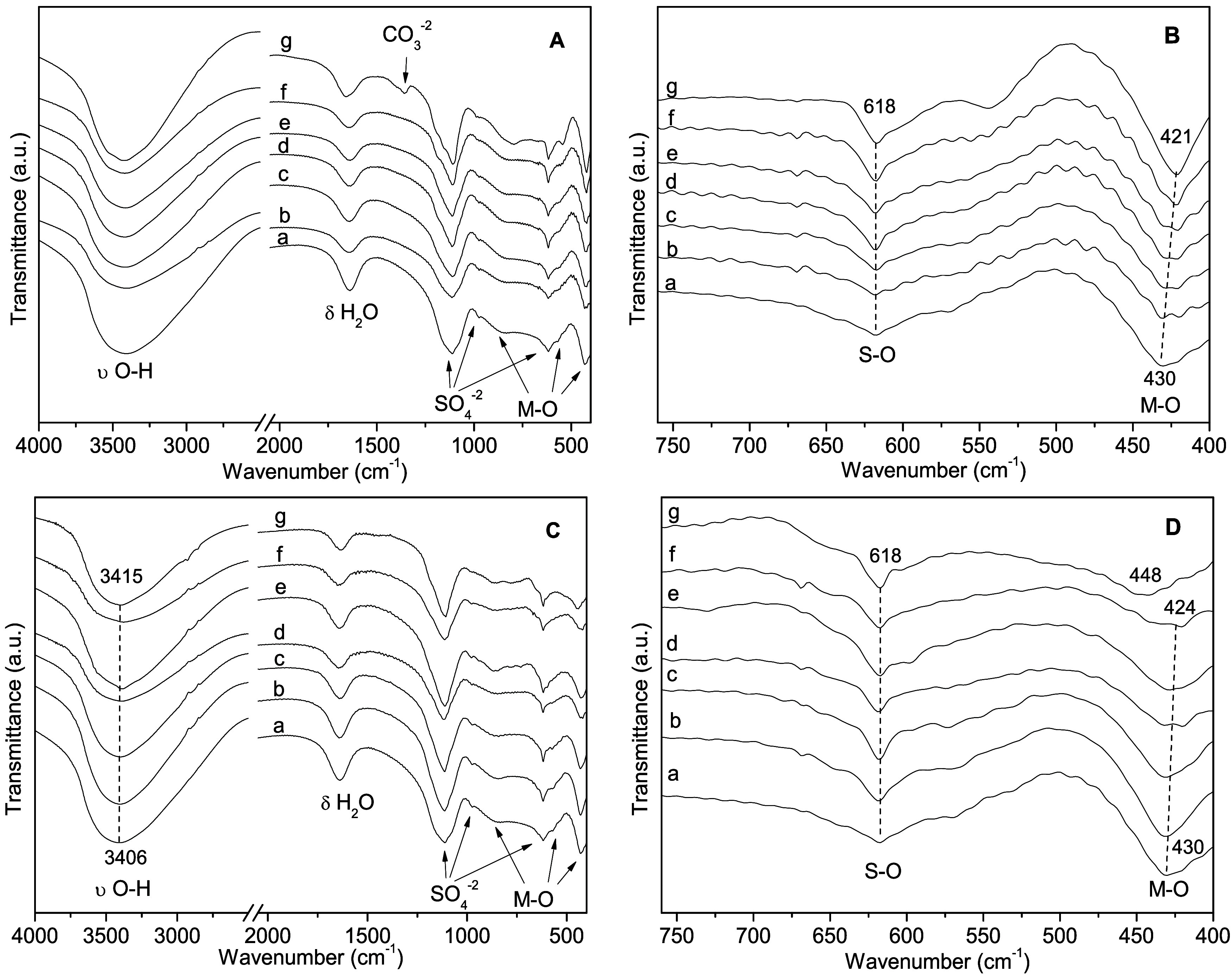
(A) FTIR spectra of the samples 6Ni-3Al (a), 5Ni/1Co-3Al (b), 4Ni/2Co-3Al (c), 3Ni/3Co-3Al (d), 2Ni/4Co-3Al (e), 1Ni/5Co-3Al (f), and 6Co-3Al (g). (B) Expansion of the FTIR spectra (A). (C) FTIR spectra of the samples 6Ni-3Al (a), 5Ni/1Cu-3Al (b), 4Ni/2Cu-3Al (c), 3Ni/3Cu-3Al (d), 2Ni/4Cu-3Al (e), 1Ni/5Cu-3Al (f), and 6Cu-3Al (g). (D) Expansion of the FTIR spectra (C).

The characteristic fingerprint bands of the S–O bond vibrations occur in the regions of 1110, 975, and 618 cm^–1^, corresponding to the asymmetric bending ν_3_, bending ν_1_ and bending ν_4_, respectively, indicating that the sulfate has a distorted tetrahedral geometry,^61,62^ similar to what has already been reported for bimetallic phases of LDHs synthesized with different M^2+^/Al compositions and intercalated with sulfate.^34,51^ For the Ni/Co group of samples (Figure 3A), the shoulder in the sulfate ν_3_ band only occurs after the addition of cobalt to the samples and becomes more prominent with the increase in the proportion of cobalt and consequent reduction of nickel. In the Ni/Cu samples (Figure 3C), this splitting of the ν_3_ band is not present, but the band is broad.

All spectra were expanded in the region from 750 to 400 cm^–1^ (Figure 3B,D) to facilitate the visualization of the shift of the band attributed to the M–O bond. For the phase containing only nickel (6Ni-3Al) (Figure 3B-a), the M–O band is located at 430 cm^–1^, and when the other metal M^+2^ is added in different proportions, the band starts moving rightward in the spectrum, located at 421 cm^–1^ for the sample containing only cobalt (6Co-3Al) (Figure 3B-g).

This shift in band position can be attributed to the M–O bond distance, with a reported bond length of 2.070 Å for Ni–O, while for Co–O, this value is slightly higher, at 2.108 Å. Longer bonds tend to have slightly weaker strengths, resulting in lower vibrational frequencies, and consequently a shift to lower wavenumbers in the FTIR spectrum.^54^ The sample synthesized with cobalt alone also showed a band at 1360 cm^–1^, attributed to contamination by carbonate ions,^63,64^ due to the pH used in the synthesis, as reported in other cobalt LDHs.^51^

For the group of samples containing copper (Figure 3C,D), based on the analysis of the 6Ni-3Al sample, the linear shift in the band related to the M–O bond is evident, which is proportional to the substitution of nickel by copper. However, in the spectrum of the sample containing only copper (6Cu-3Al) (Figure 3D-g), the vibration band of the M–O bond is shifted in the opposite direction to the others, located at 448 cm^–1^, similar to the spectra already reported for LDH samples containing Cu/Al and intercalated with sulfate obtained at different pH values.^35^

Although the Cu–O bond length (2.130 Å) is reported to be longer than that of Ni–O (2.070 Å),^54^ Cu^2+^ in an octahedral environment exhibits asymmetric distortions in the Cu–O bonds, with shorter and more rigid bonds in some directions, explaining the increase in the frequency and the shift of the band position to higher wavenumbers. Furthermore, the spectra of the samples containing Cu in different proportions (Figure 3C) also shows a small shift of the O–H band center point in the 3400 cm^–1^ region (from 3406 cm^–1^ in 6Ni-3Al to 3415 cm^–1^ in 6Cu-3Al), in addition to a small split band in the O–H band, indicating the presence of weaker and stronger bonds. This behavior is consistent with the spectra of sample complexes with benzenesulfonates and with metals used in this LDH study (Cu, Co, and Ni),^65^ in addition to octahedral complexes^66^ and different copper hydroxy-sulfates,^67,68^ which have different sizes of Cu–O bonds.

The morphological images of the Ni/Co and Ni/Cu series LDHs (Figure 4) show highly aggregated particles with submicrometric sizes. This particle agglomeration morphology occurs when the sample is prepared for analysis, usually by dripping dispersions in water over a metal sample holder until a certain mass is reached where the material can be analyzed without the electron beam reaching the metal surface. More information on the size distribution and average dimensions of the particles is unfortunately not possible to obtain by the technique used.

**Figure 4:**
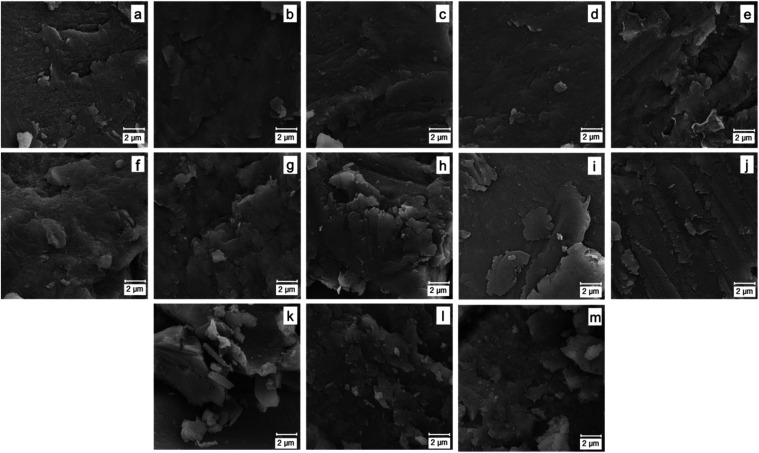
SEM images of the samples 6Ni-3Al (a), 5Ni/1Co-3Al (b), 4Ni/2Co-3Al (c), 3Ni/3Co-3Al (d), 2Ni/4Co-3Al (e), 1Ni/5Co-3Al (f), 6Co-3Al (g), 5Ni/1Cu-3Al (h), 4Ni/2Cu-3Al (i), 3Ni/3Cu-3Al (j), 2Ni/4Cu-3Al (k), 1Ni/5Cu-3Al (l), and 6Cu-3Al (m).

The sample containing only cobalt presents particles with relatively larger size in comparison to the other samples, corroborating the result obtained by XRD (Figure 1A), in which this sample presented higher crystallinity, with larger particle size and a higher number of packed layers. The EDS spectra (Figure 5) indicate the presence of all elements present in each series of compounds, attesting again to the purity of the samples.

**Figure 5:**
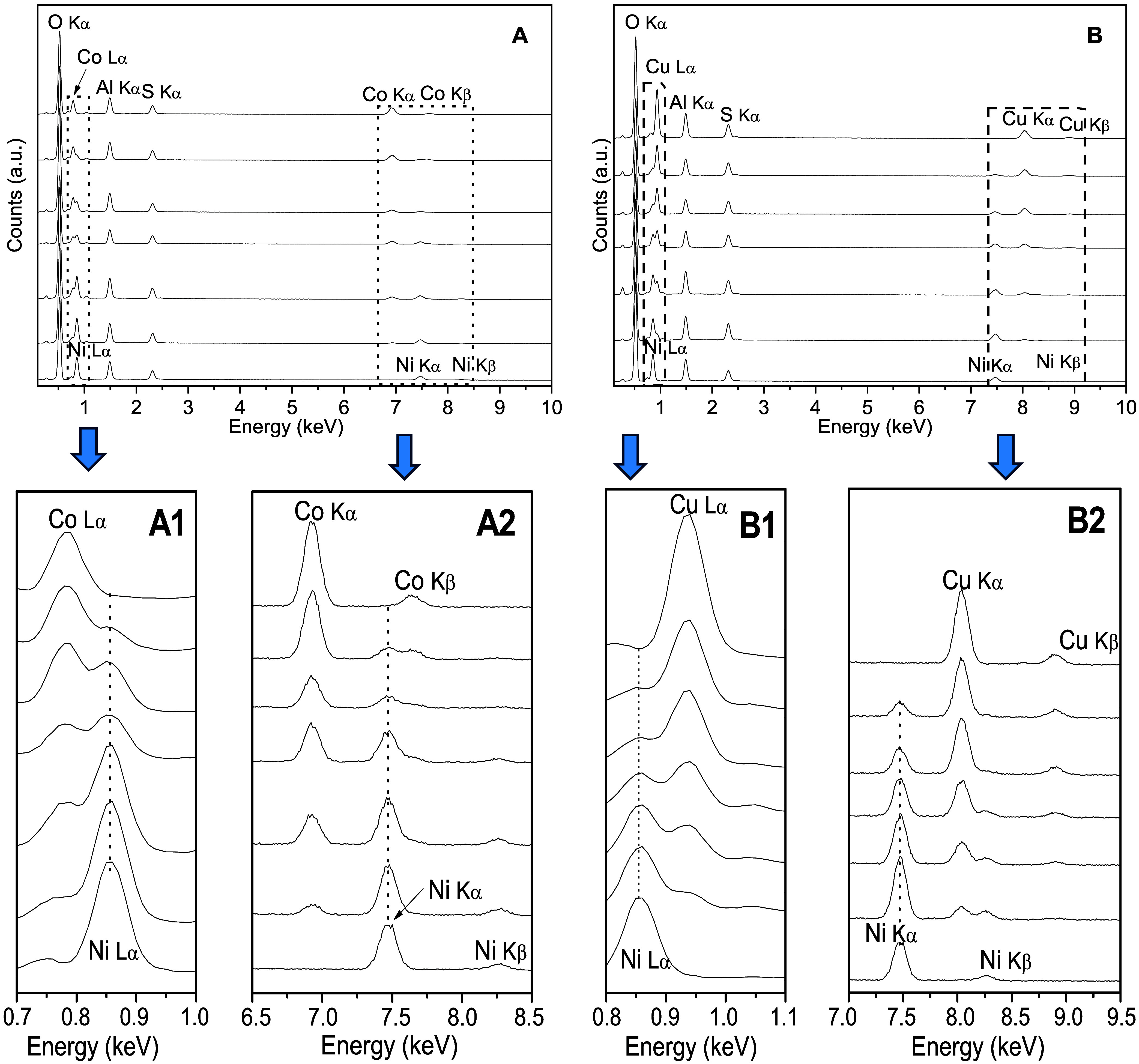
(A) EDS spectra of the samples 6Ni-3Al (a), 5Ni/1Co-3Al (b), 4Ni/2Co-3Al (c), 3Ni/3Co-3Al (d), 2Ni/4Co-3Al (e), 1Ni/5Co-3Al (f), and 6Co-3Al (g). (B) EDS spectra of the samples 6Ni-3Al (a), 5Ni/1Cu-3Al (b), 4Ni/2Cu-3Al (c), 3Ni/3Cu-3Al (d), 2Ni/4Cu-3Al (e), 1Ni/5Cu-3Al (f), and 6Cu-3Al (g). (A1, B1) Expansion of the EDS spectrum in the region of the Lα emission line of the elements Ni, Co, and Cu. (A2, B2) Expansion of the EDS spectrum in the region of the Kα and Kβ emission lines of the elements Ni, Co, and Cu.

Despite the semiquantitative analysis, it is possible to see a gradual reduction in the signals related to the characteristic Ni X-ray emission lines, located at 0.85 eV (Lα), 7.47 eV (Kα), and 8.26 eV (Kβ). This reduction is compensated by the appearance and increase of signals related to cobalt at 0.78 eV (Lα), 6.92 eV (Kα), and 7.63 eV (Kβ) as the metal is substituted (Figure 5A). The region referring to these signals is enlarged to facilitate visualization (Figure 5A1,A2).

The same occurs for the Ni/Cu sample group, with the appearance and increase in the intensity of signals related to copper at 0.93 eV (Lα), 8.03 eV (Kα), and 8.90 eV (Kβ) (Figure 5B). The results of the ICP/OES analysis of the Ni/Co and Ni/Cu LDH groups indicate that the proportion between the elements is consistent with the values used in the syntheses of the samples (Table 1) and very close to the ideal formula of the compounds [(Ni^2+^ + M^2+^)_0.666_Al_0.333_(OH)_2_]­[(SO_4_)_0.222_Na_0.111_]·H_2_O. This technique is indispensable to affirm that our materials have the formula not of a traditional LDH but of a shigaite-like LDH, where the interlayer space is occupied by sulfate anions in the presence of alkaline cations.

In general, samples from the Ni/Cu series in which impurity phases were identified (Figure 2A) also exhibited a reduced sodium content (Table 2). This is consistent with the fact that sodium is structurally associated with the shigaite-like phase, where it contributes to charge balance within the interlayer.

**Table 2:** Compositions of the Samples Obtained by ICP/OES Analyses^a^

compound	Ni^2+^	Co^2+^	Cu^2+^	Al^3+^	Na^+^	SO_4_^2–^
6Ni-3Al	0.665			0.335	0.112	0.235
5Ni/1Co-3Al	0.565	0.081		0.354	0.115	0.219
4Ni/2Co-3Al	0.447	0.220		0.333	0.108	0.239
3Ni/3Co-3Al	0.339	0.321		0.340	0.118	0.224
2Ni/4Co-3Al	0.229	0.430		0.341	0.115	0.200
1Ni/5Co-3Al	0.116	0.550		0.334	0.115	0.220
6Co-3Al		0.662		0.338	0.101	0.203
5Ni/1Cu-3Al	0.492		0.120	0.388	0.079	0.206
4Ni/2Cu-3Al	0.403		0.235	0.362	0.076	0.228
3Ni/3Cu-3Al	0.309		0.344	0.347	0.095	0.217
2Ni/4Cu-3Al	0.215		0.451	0.334	0.080	0.239
1Ni/5Cu-3Al	0.116		0.551	0.333	0.071	0.192
6Cu-3Al			0.661	0.339	0.098	0.202

a Values are based on the LDH formula: [(Ni^2+^ + M^2+^)_6_Al_3_^3+^(OH)_18_]­[(SO_4_)_2_A^+^]·12H_2_O or the reduced form [(Ni^2+^ + M^2+^)_0.666_Al_0.333_(OH)_2_]­[(SO_4_)_0.222_Na_0.111_]·H_2_O. Indices in the formula are 1 = 0.111; 2 = 0.222; 3 = 0.333; 4 = 0.444; 5 = 0.555; 6 = 0.666.

A decrease in sodium content therefore suggests a deviation from the ideal shigaite-like structure and the possible formation of secondary hydrotalcite-like phases where sodium is absent or other amorphous non-LDH phases such as Al­(OH)_3_ or copper-containing oxides/carbonates. These secondary phases are not expected to host sodium in the same manner, which explains the observed compositional shifts.

## Antibacterial Activity

The agar diffusion method with bacteria allows the evaluation of the antimicrobial activity of a compound by testing the ability of active species, in this case the bimetallic and trimetallic LDHs, to diffuse through a solid agar medium, inhibiting bacterial growth in a circular zone called the inhibition zone. The inhibition zones of the Gram-positive *S. aureus* and Gram-negative *E. coli*, obtained for the bimetallic LDHs 6Ni-3Al, 6Co-3Al, and 6Cu-3Al, as well as for the intermediate trimetallic LDHs 3Ni/3Co-3Al and 3Ni/3Cu-3Al, are shown in Figure 6 and Table 3.

**Figure 6:**
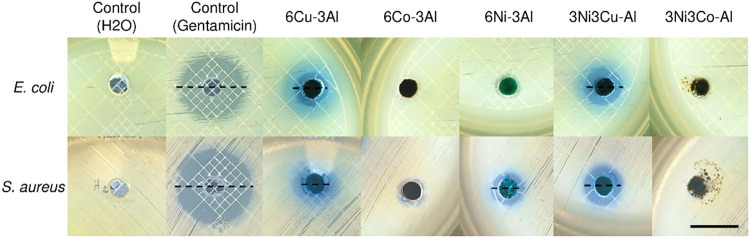
Photographs of agar plates inoculated with *E. coli* and *S. aureus* after 24 h of incubation (scale bar: 20 mm).

**Table 3:** Inhibition Zones for *E. coli* and *S. aureus* in Each of the LDHs Studied after 24 h of Incubation

	*E. coli*	*S. aureus*
H_2_O		
gentamicin	27.5 ± 1.5	33.5 ± 2.5
6Cu-3Al	13.0 ± 1.0	13.5 ± 0.5
6Co-3Al		
6Ni-3Al		
3Ni3Cu-3Al	14.0 ± 2.0	14.0 ± 1.0
3Ni3Co-3Al		

The LDHs containing copper (6Cu-3Al and 3Ni/3Cu-3Al) exhibited a pronounced antibacterial effect against both *E. coli* and *S. aureus*. After 24 h of incubation, inhibition zones of 13 ± 1.0 mm (6Cu-3Al) and 14 ± 2.0 mm (3Ni/3Cu-3Al) were obtained for *E. coli*, whereas for *S. aureus*, the zones reached 13.5 ± 0.5 mm (6Cu-3Al) and 14 ± 1.0 mm (3Ni/3Cu-3Al). Supporting Figure S1 presents magnified views of the inhibition halos for each investigated material, including the corresponding halo diameter measurements. Supporting Figure S2 shows the complete Petri dishes without magnification, with the inhibition halos identified and highlighted to facilitate visualization and comparison between the tested samples.

As the copper-containing sample (6Cu-3Al) exhibited inhibition zones against both Gram-negative and Gram-positive bacteria, the results are consistent with previous findings for a Ca/Al LDH intercalated with a copper complex, which showed higher inhibition efficiency against *E. coli* compared to a cobalt-intercalated analogue, highlighting the antibacterial activity of copper ions.^69^ Consistent with this finding, the 6Co-3Al sample had no inhibitory effect against either *E. coli* or *S. aureus*.

A partial substitution of Ni by Cu cations improved the inhibition zones of LDH powders, which may be attributed to the chemical nature of the divalent cation. A previous study on trimetallic Mg/Cu–Al LDHs reported that increasing the copper content generally enhanced antibacterial activity, leading to the appearance or enlargement of inhibition zones against several strains, including *Enterococcus faecalis*, *Bacillus subtilis*, *E. coli*, *S. aureus*, and *Pseudomonas aeruginosa*.^70^

The exact antibacterial mechanism of LDHs is complex and involves multiple factors, including the bacterial type (*E. coli* or *S. aureus* in this study) as well as the physicochemical characteristics of the LDH particles. Although the bimetallic LDHs (CuAl, CoAl, and NiAl) were all synthesized using the same coprecipitation procedure, the distinct ionic radii and coordination geometries of the divalent cations influence their incorporation into the layered structure. Other amorphous copper compounds in low concentration may be present as impurities in the LDH and may contribute to the release of active Cu^2+^ species.

The release of divalent cations from the LDH structures into saline solution is summarized in Table 4. For the 6Ni–3Al sample, the initial Ni^2+^ content was 0.4982, with a gradual increase in released concentration over time, reaching 0.0008 (0.16 wt %) after 1 h, 0.0011 (0.22 wt %) after 24 h, and 0.0019 (0.38 wt %) after 7 days. A similar trend was observed for the 6Cu–3Al sample, with an initial Cu^2+^ content of 0.8815 and released amounts of 0.0017 (0.19 wt %) after 1 h, 0.0037 (0.42 wt %) after 24 h, and 0.0041 (0.47 wt %) after 7 days.

**Table 4:** Release of Ni^2+^ and Cu^2+^ from 6Ni-3Al and 6Cu-3Al

compound	time	M^2+^ (Ni or Cu)
6Ni-3Al	initial (0 h)	0.4982
	1 h	0.0008 (0.16 wt %)
	24 h	0.0011 (0.22 wt %)
	7 days	0.0019 (0.38 wt %)
6Cu-3Al	initial (0 h)	0.8815
	1 h	0.0017 (0.19 wt %)
	24 h	0.0037 (0.42 wt %)
	7 days	0.0041 (0.47 wt %)

These results indicate a progressive and relatively limited release of metal cations from the LDH structure over time, suggesting controlled dissolution behavior. Although the total released Ni^2+^ and Cu^2+^ remains below 0.5 wt % after 7 days, even low concentrations of dissolved copper ions may contribute to antibacterial activity.

The role of Cu^2+^ ions in disrupting bacterial cell membranes and metabolic processes are well documented in the literature. Therefore, while the present data do not allow a definitive determination of the possible antibacterial mechanisms, they support a correlation between the nature of the ion released and the observed inhibition of bacterial growth. This is consistent with the results obtained from agar diffusion assays, where the antibacterial effect is likely governed by the diffusion of released ionic species rather than direct particle–cell interactions.

## Conclusions

In this work, a series of bimetallic and trimetallic layered double hydroxides (LDHs) based on Ni, Co, and Cu were successfully synthesized by coprecipitation under controlled pH conditions, leading to materials with compositions close to the expected shigaite-like structure. Structural and compositional characterization confirmed the formation of layered compounds with typical platelet-like morphology and variations in crystallinity depending on the metal composition.

The antibacterial evaluation, performed using agar diffusion assays, showed that Cu-containing LDHs exhibited higher activity against *S. aureus* and *E. coli* compared with Ni- and Co-based systems. Considering the limited solubility of these materials, this effect is most likely associated with the release of Cu^2+^ ions, although the definitive inhibition mechanism was not identified.

These results indicate that LDHs containing copper are promising systems for antibacterial applications, particularly because of their ability to act as reservoirs for the gradual release of active species and to accommodate additional functional anions in the interlayer space. To better understand the effect of these materials on different bacterial systems, further studies based on quantitative assays and more detailed ion-release analyses are still required.

## Supplementary Material



## Notes

Calculated values of particle size and average number of packed layers obtained using the Scherrer equation; photographs of inhibition zones for *E. coli* and *S. aureus* after 24 h of incubation; and complete Petri dish images containing the investigated samples against *E. coli* and *S. aureus* (PDF)
