# Supplementary material for: Antibacterial Activity of New Shigaite-like Ni/Co–Al and Ni/Cu–Al Layered Double Hydroxides Intercalated with Sulfate and Sodium Cations: Preliminary Studies
Source: ACS Omega. 2026 Jun 12;11(25):37818–28. doi: 10.1021/acsomega.6c02816 (PMC13325139; doi:10.1021/acsomega.6c02816)
Supplement: Supplementary file 1 [file ao6c02816_si_001.pdf]

Supplementary Material

Antibacterial activity of new shigaite-like Ni/Co-Al and Ni/Cu-Al layered double hydroxides, intercalated with sulfate and sodium cations: preliminary studies

Anne Raquel Sotiles[1]*, Vitor Vianna de Souza Machado[1,2], Monica Surek[3], Cláudia Eliana Bruno Marino[2], Fernando Wypych[1,4]*

[1] Department of Chemistry, Centro Politécnico, Federal University of Paraná, CP 19032, Jardim das Américas, 81531-980, Curitiba, Paraná, Brazil
[2] Department of Mechanical Engineering, Program in Engineering and Materials Science (PIPE), Centro Politécnico, Federal University of Paraná, CP 19011, Jardim das Américas, 81531-980, Curitiba, Paraná, Brazil
[3] Department of Clinical Analysis, Campus Jardim Botânico, Federal University of Paraná, Jardim Botânico, 80210-170, Curitiba, Paraná, Brazil
[4] Federal University of Technology – Paraná, Avenida dos Pioneiros, 3131, 86036-370, Londrina, Paraná, Brazil

Supplementary material 1 - Calculated values of particle size and average number of packed layers, using the Scherrer equation.

Compound	Crystalline domain size along the basal direction (nm)	Average number of packed layers
6Ni-3Al	4.75	4.37
5Ni/1Co-3Al	4.62	4.29
4Ni/2Co-3Al	4.45	4.13
3Ni/3Co-3Al	4.91	4.56
2Ni/4Co-3Al	4.78	4.42
1Ni/5Co-3Al	6.85	6.23
6Co-3Al	14.64	13.17
5Ni/1Cu-3Al	4.41	4.17
4Ni/2Cu-3Al	4.67	4.39
3Ni/3Cu-3Al	4.62	4.34
2Ni/4Cu-3Al	5.52	5.19
1Ni/5Cu-3Al	6.30	5.90
6Cu-3Al	10.12	9.43

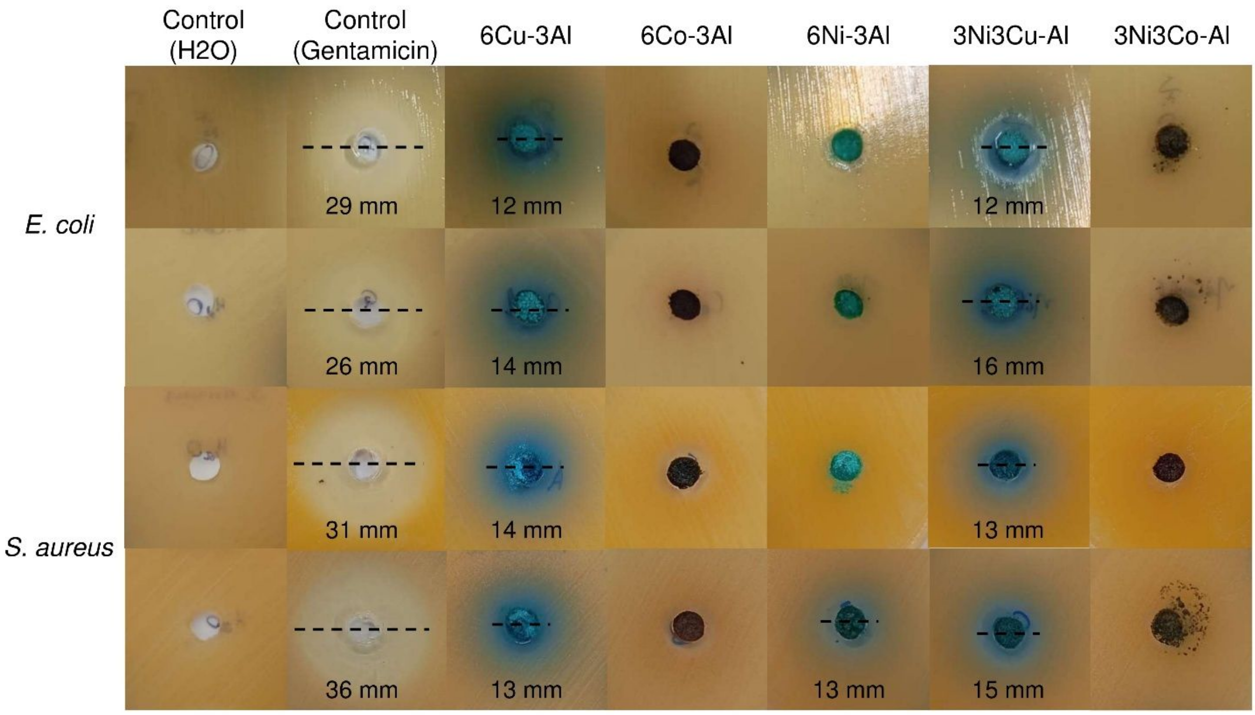

Figure S1 - Pictures of replicates for *E. coli* and *S. aureus* inhibition zones from all samples after 24 h of incubation.

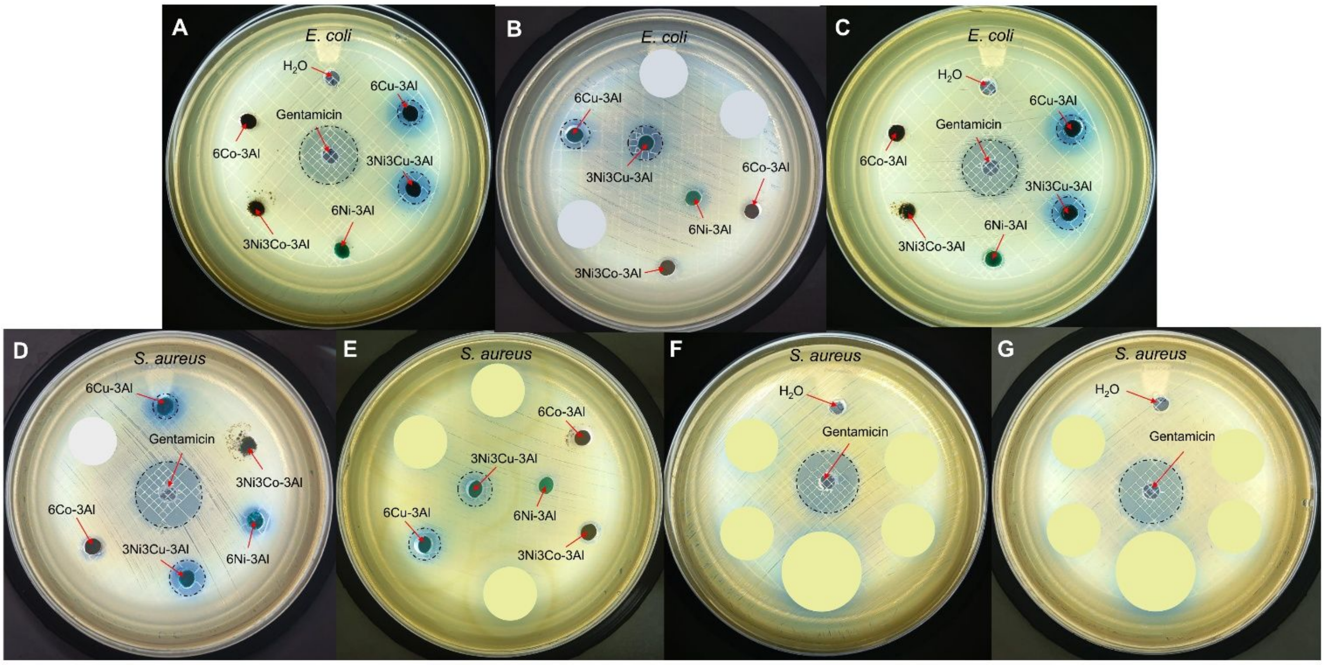

Figure S2 - Complete Petri dishes containing the samples investigated in this study against (A–C) E. coli and (D–G) S. aureus colonies. Visible inhibition halos surrounding some samples indicate bacterial growth suppression, whereas the absence of halo formation suggests limited antibacterial activity under the evaluated conditions. Significant inhibition zones were observed mainly for 6Cu-3Al and 3Ni3Cu-3Al containing samples, while 6Co-3Al, 3Ni3Co-3Al, and 6Ni-3Al samples showed little or no inhibition against either bacterial strain. Filled circles indicate regions corresponding to additional samples originally present in the Petri dishes but not discussed in the present work. These regions were masked solely to improve visualization and maintain focus on the samples investigated in this manuscript.
